# Interpersonal and intrapersonal leadership competencies: an interpretative phenomenological analysis on effective leadership strategies

**DOI:** 10.3389/fpsyg.2025.1553620

**Published:** 2025-08-20

**Authors:** Marion Koreen Andrea Aquino, Alicia Mae Bumacod, Arizzandra Gerriz Calapine, Jan Marie Therese Yaco, Margarett Luz Dimailig, Avon Pearl Amores, Joseph Francisco

**Affiliations:** People Dynamics, Inc., Pasig, Philippines

**Keywords:** effective leader, interpersonal competency, intrapersonal competency, interpretative phenomenological analysis, organizational leadership

## Abstract

**Introduction:**

In today’s dynamic business environment, unforeseen challenges have become increasingly prevalent as markets evolve. Effective leadership is critical to organizational success, requiring leaders who can efficiently manage both their tasks and employees. Thus, it is valuable to explore and identify competencies possessed by competent leaders.

**Methods:**

The study utilized an Interpretative Phenomenological Analysis (IPA) approach to have a detailed exploration of the strategies and practices leaders find most effective in navigating complex organizational challenges, such as strategy adoption, teamwork improvement, client relationship management, ethical decision-making, and industry adaptation. Purposive sampling was used to select five Filipino organizational leaders from diverse industries. The small yet varied sample allowed for an in-depth analysis of personal experiences while ensuring that the findings resonate broadly within the Philippine organizational context. Data collection involved in-depth, semi-structured interviews conducted via Microsoft Teams. The interviews were transcribed, coded, and thematically analyzed in accordance with IPA guidelines.

**Results and discussion:**

Competencies were categorized under the five competency areas, with further exploration of interpersonal and intrapersonal impacts. The iterative analysis revealed recurring competencies across all areas. Intrapersonal competencies included personal growth through change, leadership through vision and resilience, continuous learning, innovative thinking and openness, and resilience in personal challenges. Meanwhile, interpersonal competencies included collective innovation, effective communication, transformative client engagement, empathetic conflict resolution, and building adaptive teams. These enabled leaders to maintain their competence in navigating organizational challenges, including the pandemic, technological advancements, and evolving workforce dynamics. These findings suggest that effective leadership operates on both individual and interpersonal levels. Leaders’ internal processes significantly influence their relationships and interactions. Resilient leaders stabilize teams during crises, while a commitment to continuous learning fosters innovation and collaboration. Strong communication skills promote knowledge sharing and strengthen relationships with clients, subordinates, and stakeholders. Empathetic leadership reduces stress and conflict, creating healthier work environments. Finally, leaders’ motivation and dedication inspire team transformation and adaptability. Thus, these leadership competencies are instrumental in sustaining a competitive edge and driving organizational growth amidst a dynamic business environment.

## 1 Introduction

Effective leadership, motivated by strong interpersonal and intrapersonal competence, is crucial for organizational success. Organizational leadership involves guiding members toward shared goals and supporting them through challenges and growth (Saeidi et al., 2021). Interpersonal competence is the ability of leaders to achieve team and personal goals while maintaining positive relationships with the members (Shek and Lin, 2015). In the same study, intrapersonal competence encompasses a leader’s internal processes and internalization of high-performance standards. Effective leadership requires a balance of interpersonal and intrapersonal skills to drive team performance and organizational success.

Although widely discussed, the application of these competencies in the Philippine context remains unexplored. Examining further how Filipino leaders apply them in real-world settings could help researchers gain insights into navigating key aspects of their organizational leadership roles by addressing the following questions:

1. How do leaders experience and interpret the process of adopting new strategies within their departments?
2. How do leaders perceive and describe their efforts to enhance teamwork quality?
3. What are the lived experiences of leaders in building and maintaining strong client relationships, and how do they handle the termination of these relationships?
4. How do leaders experience and navigate ethical dilemmas in their decision-making processes?
5. How do leaders adapt to changing industry environments, and what strategies do they find compelling in managing these changes?

Although global literature offers insights into leadership competencies, it remains primarily grounded in Western, industrialized contexts. Highlighting universal principles, it often overlooks how culturally distinct environments shape leadership competencies. As leadership is not culturally neutral, one practice may be effective in a particular country but inappropriate in another (Vander Pal and Ko, 2014). This gap underscores the need to explore leadership within non-Western, developing contexts.

Leadership development improves when supervisors support autonomy by encouraging choice, recognizing emotions, and avoiding coercion, leading even lower-ranking employees to feel more empowered and self-directed (Slemp et al., 2018). In contrast, many Filipino organizations use top-down decisions, limiting employee’s autonomy (Mapoy et al., 2021). This dynamic is often rooted in local cultural norms that favor discipline, hierarchy, and indirect communication.

Furthermore, as a developing country, the Philippines faces structural challenges in leadership development, including limited access to comprehensive training programs (Jordan, 2024). Cultural values also influence how leaders resolve conflict, communicate, and make decisions. In collectivistic societies such as the Philippines (Datu and Reyes, 2015), indirect strategies like silent resistance, going through intermediaries, or waiting for tensions to subside are often seen not as weaknesses but as culturally appropriate and practical approaches to maintaining group harmony (Vander Pal and Ko, 2014).

The rise of global leadership highlights cultural distinctions that shape organizational practices. Given these gaps, it becomes imperative to examine how Filipino leaders apply competencies such as strategy adoption, teamwork quality, client relationship management, ethical decision-making, and industry adaptation, as it is often overlooked in a universal context. This study examines how they navigate interpersonal and intrapersonal competencies in the context of real-world, culturally embedded organizational challenges.

## 2 Literature review

The first key leadership area is strategy adoption. Leaders implement new strategies and guide teams through change. Hussain et al. (2018) identified leadership, employee involvement, and knowledge sharing as key change factors. During transitions, leaders must offer support, incentives, and clear guidance (Hussain et al., 2018, as cited in Musaigwa, 2023). Communication should explain role impacts, align with company values, clarify goals, and outline resources (Yue et al., 2020). Adaptable leaders foster innovation, learning, and team effectiveness amid constant change (Stehnkuhl, 2024).

Secondly, teamwork quality is another key area of responsibility for a leader. Leaders foster strong team dynamics by resolving internal and external issues (Zhao et al., 2022). Effective teamwork involves collaboration, idea exchange, and mutual support (Fathin and Raharjo, 2023). Building trust and harmonious communication is essential (Luca and Tarricone, 2002, as cited in Dhurup et al., 2016). Ethical behavior, leadership, communication, teamwork skills, and emotional intelligence are crucial for managing relationships and promoting positive dynamics (Mercader et al., 2021; Eva et al., 2024; Kasrah et al., 2024). This competence boosts productivity and success.

The third key area emphasizes leaders’ proficiency in cultivating robust client relationships while steering teams toward strategic objectives. Client relationship management involves building and maintaining relationships to create value for both client and business (Boljanovic and Stankovic, 2012, as cited in Oksana, 2018). Understanding client needs and the manager’s role is essential (Tahan, 2018). Competencies like emotional intelligence, leadership performance, and teamwork predict client satisfaction (Adu, 2020). This requires empathy, strong leadership, and strategic customer knowledge management.

The fourth key area is a leader’s ability to navigate ethical challenges while serving as a role model (Ko et al., 2018). Ethical decision-making is a core of effective leadership. Ethical leaders, with moral awareness and an employee-focused approach, foster ethical workplaces (Touma, 2022). This promotes employee growth through interpersonal justice and clear rules (Rahaman et al., 2021). They also self-regulate, maintain integrity, and cope using intrapersonal skills (Merlin and Soubramanian, 2024). Ethical decision-making remains essential in evaluating leadership.

Lastly, industry adaptation assesses leaders’ ability to adjust to changes from the pandemic and technological advances (Goniewicz and Hertelendy, 2023). Stefan and Nazarov (2020) emphasized considering work schedules, team dynamics, health, and job requirements when assigning tasks. Transformational leadership supports change and employee training (Faupel and Süß, 2019). Interpersonal and intrapersonal competencies help leaders manage stress, build diverse teams, and foster taking a team perspective (Schulze and Pinkow, 2020; Marbun, 2021). These studies show the importance of leaders’ adaptability when dealing with changes.

Recent research in the Philippine service industry highlights the importance of leadership competencies in managing a multigenerational workforce and fostering motivation and positive culture (Kurata et al., 2022). Hamilton and Kuchinka (2022) found Filipino-American leaders balance assertiveness with traditional values, a skill essential for strategy adoption. These findings underscore adaptability’s role in responding to organizational and industry changes.

Filipino leaders now prioritize relationship-building and harmonious workplaces, especially in multicultural settings, to build long-term client ties (Dequilla et al., 2023). Ethical leadership balances goals with employee wellbeing and trust (Lantican, 2020). Digital transformation reshapes leadership as technology enhances operations and customer experience, a shift accelerated by COVID-19 (Caringal-Go et al., 2024). Agile leadership, with decentralized decision-making, also improves efficiency and responsiveness (Luningning, 2023).

In sum, the cited studies underscore the multifaceted nature of leadership competencies, which are applicable in the modern dynamics and culture of a diverse work environment. Across all five key areas, both interpersonal and intrapersonal competencies are necessary. Effective leadership requires adaptability, ethical integrity, cultural sensitivity, and strong relationship-building to manage change and drive organizational success.

## 3 Materials and methods

Over the years, scholars have tried to develop numerous ideas to explain what constitutes effective leadership, thus, becomes central to the inquiry process of the researchers of this study. Leadership is not merely a set of overarching overt set of behaviors, as cited by Miller and Sardais (2011), it includes an interplay of interpersonal dynamics, individual cognitive processes, personality differences, and the subjective meaning individuals ascribe to their personal experiences. Therefore, studying leadership, as seen from the studies conducted by Arda et al. (2016), necessitates a research approach that can capture the intricate layers of human experience, revealing the full complexity of it must be supported by a methodological approach that can see the subtle nuances of leadership styles (Safonov et al., 2018).

Interpretative Phenomenological Analysis (IPA) was chosen for this research because it offers a robust framework to explore how individuals make sense of their personal and professional experiences. The works of Arda et al. (2016) and Safonov et al. (2018) support the need for qualitative methods that can reveal the depth and subtlety of leadership as experienced by those in leadership roles. IPA aligns with this objective by placing the individual’s perspective at the center of inquiry. Central to the IPA approach is its focus on exploring in-depth analysis of one’s individual lived experiences, making it highly distinguished among other qualitative methods. This triadic framework enables researchers to analyze the internal processes behind overt leadership skills. IPA allows the researchers to convey participants’ own understanding of their competencies and contextually grounding their experiences without fixing it in a static construct. This method also supports a more humanistic and dynamic approach of leadership skills rather than treating it as mere objective variables.

In addition, the robust framework of IPA provides an avenue to explore the rich contribution of the combined ideas of phenomenology, idiography and the dualistic hermeneutics that converge into a rich tapestry of human experiences. It also empowers the researchers to gain traction of profound insights of the participants, especially how leaders interpret their own experiences and make sense of the unique interaction within their department, as they acknowledge a multifaceted leadership styles that happen within could also be transcendental looking from inside to outside perspective. To add, given that both interpersonal and intrapersonal dynamics in an organization, are greatly anchored in the individual’s perceptions and subjective interpretations, the said approach makes it a powerful lens to explore different enacted facets in leadership, such as, communication, empathy, conflict resolution and even one’s views about the uniqueness of any given organizational culture.

### 3.1 Sample

Since IPA is a rigorous and in-depth qualitative methodology, its implementation requires specific strategies as recommended by Smith et al. (2009). One of these is the use of a small sample size—typically fewer than 10 participants—to allow the researcher to deeply engage with each individual’s experience on a case-by-case basis (Pietkiewicz and Smith, 2014). Given IPA’s idiographic nature, a smaller sample ensures that each participant’s voice is clearly represented within the study.

As stated in the introduction of the methodology section, leadership is a complex and multifaceted phenomenon. It cannot be reduced to a singular leadership style, as it involves internal cognitive processes, interpersonal dynamics, organizational culture, and broader sociocultural contexts. The temporal dimension—when and in what context leadership experiences occurred—also plays a crucial role. Due to these layers, the analysis must be deep and detailed for each individual. Therefore, our research aligns with Noon’s (2018) study, which also involved only five interviewees to avoid data overload. This approach enabled the researcher to focus exclusively on each participant and thoroughly explore their internal, relational, cultural, temporal, and organizational contexts.

This does not suggest, however, that our dataset lacked variability. As Tracy (2020) emphasized, the quality of qualitative research is not solely determined by the sample size but also by the relevance and diversity of the participants. In line with the recommendations of Tracy (2020) and Subedi (2021), while all participants in this study shared the common experience of being organizational leaders, they were selected from varied functions and domains. Though all were employed by the same company, the organization’s diverse service offerings allowed the study to draw from two distinct industries: industrial assessment services and business consulting.

During participant recruitment, the researchers reached out via email to organizational leaders within the company. The participants represented various functional areas, including sales, human resources, assessment services, administrative services, business development, quality assurance, and business consulting. Only departments with individuals who gave informed consent were included, ensuring that participants were willing to offer genuine insights aligned with the study’s goals.

Since these participants came from different fields and industries, they were able to provide diverse insights shaped by their unique work contexts and demands. While all participants experienced similar leadership challenges—such as persuading subordinates, enhancing teamwork, managing client relationships, navigating ethical decisions, and adapting to industry changes—the nuances of how these challenges were encountered differed depending on their specific context.

As supported by Chan (2010), leadership development is shaped more by the reflective processing of experience than by mere tenure. Although this study included only those with at least 2 years of accumulated supervisory or managerial experience, the focus remained on capturing the depth of each leader’s reflections rather than quantifying experience by years alone.

In sum, this sampling approach enabled the researchers to explore participants’ leadership strategies in depth, while also investigating the internal, relational, organizational, and cultural dynamics surrounding these strategies. This allowed IPA to be fully utilized in providing rich, nuanced, and contextualized interpretations of each participant’s lived experience (Brocki and Wearden, 2006). This purposive sampling, therefore, captures the complexity of leadership experiences and ensures that the insights gathered resonate broadly within the Filipino organizational landscape, reinforcing the study’s contribution to the field of leadership competencies.

### 3.2 Researchers’ characteristics and reflexivity

The researchers, employed as licensed psychometricians and psychologists in a company that develops standardized industrial assessments, possess prior knowledge of leadership competencies through literature reviews conducted during the development of tools for supervisory and managerial roles.

For practical reasons, participants were selected from the same organization. As a result, a degree of professional familiarity existed. The researchers were aware of the participants’ roles, designations, and functions within the company, including identifying the department heads and understanding the organizational culture. However, this familiarity did not involve direct engagement, supervision, or personal relationships. The researchers had no close interpersonal ties with participants; the interaction remained neutral and professional.

To further mitigate bias and ensure consistency, only one researcher, who also served as the interviewer and primary reviewer of data, interacted directly with participants throughout the entire data collection and member checking process. This helped maintain uniformity in how questions were asked, how responses were followed up, and how clarifications were sought. The rest of the research team deliberately limited their contact with the participants and were assigned to review data from individuals with whom they had no personal connection, reinforcing neutrality.

This professional familiarity proved advantageous as it facilitated smoother recruitment, quicker management approval, and increased participant cooperation (Berger, 2015), without compromising objectivity or introducing bias. Furthermore, the researchers’ contextual understanding of internal policies and leadership practices facilitated the accurate interpretation of participant narratives. The familiarity also supported rapport-building and effective communication, which are essential components in any form of qualitative interviewing (Roiha and Iikkanen, 2022, as cited in Roiha et al., 2024), but only to the extent of encouraging open and comfortable dialogue.

This dynamic, while aiding in the richness of responses, remained strictly within professional boundaries. As such, this alignment with IPA’s principles enabled the collection of rich, contextualized testimonies while upholding methodological integrity.

### 3.3 Ethical consideration

As part of research ethics, an informed consent form was provided to participants several days before the interviews. The form, seen in Appendix, detailed the study’s purpose, procedures, potential risks, benefits, and data confidentiality measures. Participants were informed the interviews aimed to explore leadership experiences to develop a leadership skills assessment tool.

Those who agreed to take part were asked to sign a consent form and were provided with interview questions in advance. Although the study followed an IPA approach, the questions were shared beforehand to promote transparency, reduce potential anxiety, and allow participants to reflect more deeply on their experiences. This preparatory step helped ensure participant comfort and richer responses, without compromising the flexibility of the interview process, as follow-up questions and the exploration of emergent themes remained integral to the IPA methodology.

### 3.4 Data gathering

The study employed in-depth, semi-structured interviews, deliberately chosen to align with its objectives and IPA, which aims to explore participants’ testimonies in depth. With interviews, researchers are able to facilitate participants to reflect on their experiences as organizational leaders (Smith et al., 2009). Since the participants were employed in the same company as the researchers, a one-on-one interview approach was chosen when designing the interview schedule. Group interviews were deemed inappropriate, as participants might feel uncomfortable discussing personal workplace experiences in the presence of colleagues. Following Noon’s (2018) recommendation, the interviews were conducted privately via Microsoft Teams, with only the participant and the sole interviewer present. This setup ensured confidentiality and created a safe space for participants to share openly, without fear of judgment or potential repercussions from management. As a result, the researcher was able to establish rapport and facilitate the elicitation of more intimate, in-depth accounts, with the focus solely on the individual being interviewed.

To minimize bias and ensure consistency in data collection, the researchers developed a structured interview protocol. While IPA traditionally emphasizes open exploration of lived experiences, the inclusion of five pre-identified leadership competencies was intentional and strategic. Drawn from prior empirical studies and theoretical frameworks, these competencies served as an anchor to guide the interviews toward areas directly relevant to the research questions. Similar to Noon’s (2018) and Smith’s (2015) funneling technique, this approach allowed the interviews to maintain a clear focus on competencies vital for organizational leadership while still providing space for participants to share individual experiences and introduce emergent themes beyond the predefined structure. The interview questions were carefully crafted to align with this competency framework and to elicit rich, experiential insights into the interpersonal and intrapersonal aspects of leadership. The following questions in Table 1 served as the core focus of the Main Interview.

**Table 1:** Core interview questions and their objectives.

Interview question	Justification
Think of a time when you had to fix an internal issue regarding your department’s teamwork quality. What is a good strategy for this? What are the things that should be avoided when handling this?	The first question, “Strategy Adoption,” explores leaders’ persuasive abilities and approaches to implementing strategies. By examining effective and ineffective methods, this question sheds light on leaders’ capacities to secure departmental alignment with strategic goals.
Think of a time when you had to convince your department to go with your strategy. What is a good way to handle this? What is a bad way to go about this?	In examining Teamwork Quality, the second question delves into how leaders resolve internal team issues. This question emphasizes problem-solving and collaboration skills, focusing on the interpersonal competencies necessary for fostering a cohesive team dynamic while identifying commonly encountered challenges.
Think of a time when you had to maintain a good relationship with a customer. How do you ensure their loyalty as the company’s client? Was there a time when a relationship with a client was terminated?	The third question concerns Client Relationship Management, targeting the interpersonal and emotional intelligence skills that leaders employ to build and sustain client loyalty. This question provides a nuanced view of how leaders manage client interactions and loyalty by including scenarios involving sustained and terminated client relationships.
Think of a time when you faced a dilemma choosing between an ethical solution and a more practical solution. How did you handle this?	The fourth question, aligned with Ethical Decision-Making, explores leaders’ approaches to navigating ethical dilemmas where practical solutions may conflict with ethical standards. This question allows insights into leaders’ moral reasoning and integrity as they balance organizational goals with ethical principles.
Think of a time when you had to deal with the changing environment of your business’ industry. How did you address this?	Lastly, Industry Adaptation is assessed through a question that investigates leaders’ responses to industry changes. This question highlights adaptive competencies such as flexibility, resilience, and strategic thinking, providing a view into how leaders navigate and manage their industry’s evolving demands.

### 3.5 Validity and reliability

Since the researchers had a degree of professional familiarity with the participants, multiple strategies were employed to minimize bias and ensure the validity and reliability of the data. Given that IPA, both the researcher and participant aim to make sense of the participant’s lived experience, it is essential to maintain transparent documentation of the analytical decision-making process. Following the recommendation of Smith et al. (2009), the researchers utilized an Excel-based audit trail and reflective journal to capture their evolving interpretations and decisions throughout the analysis.

This file was regularly revisited, allowing the researchers to re-read interview transcripts alongside their reflective entries. This practice ensured that interpretations remained closely grounded in the participants’ actual responses, supporting a dynamic and iterative approach to theme development.

**Figure 1:**
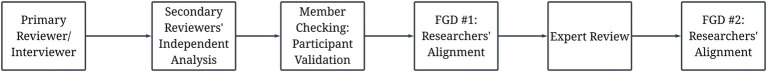
Triangulation process.

The audit trail also played a central role in investigator triangulation (Carter et al., 2014), as seen in Figure 1. The researchers served as each other’s reviewers. The primary reviewer, who conducted all the interviews, produced the first round of analyses. The secondary reviewers then cross-checked the transcripts, conducted their independent analyses of the recordings, and compared findings. An expert review was conducted by a licensed psychologist, who then evaluated the results of the initial analysis. The use of the audit trail enabled the researchers to trace the development of themes at any stage of the process, ensuring methodological rigor and transparency.

A focus group discussion (FGD) was also conducted to identify and reflect on potential personal biases or assumptions that may have influenced the interpretations. This session provided a structured opportunity for the research team to discuss their thought processes, align thematic insights, and collaboratively refine the findings.

Lastly, member checking was integral to the process (Dimler et al., 2017). Participants were re-invited to review the preliminary findings, during which they were presented with verbatim transcriptions alongside the researchers’ initial analyses to confirm accuracy and determine whether the interpretations genuinely reflected their lived experiences.

In summary, this approach established a systematic process of participant and peer triangulation, allowing the researchers to accurately capture the participants’ perspectives while continuously refining the emerging themes, thus ensuring reliability and validity of the research.

### 3.6 Data analysis

The data analysis employed in this study followed a multiperspective, group-level IPA (Larkin et al., 2019). While grounded in the core principles of IPA (Smith et al., 2009), the approach also considered shared experiences across participants, as seen in Table 2.

**Table 2:** Step-by-step IPA procedure.

Step number	Process	Description	Source
*Step 1*. Reading and re-reading	Reviewers immerse themselves in the raw data to accurately interpret the participants’ experiences.	The primary reviewer/interviewer transcribed the interviews in Microsoft Word, capturing verbatim responses and notable nonverbal cues such as laughter. Once completed, secondary reviewers cross-checked the transcriptions against the recordings to ensure accuracy and consistency.	Smith et al. (2009)
*Step 2*. Initial noting	Condense the words in a transcript		
	Descriptive comments	Reviewers outlined the experience antecedents, the participant’s behavior, and the resulting consequences.	
	Linguistic comments	Reviewers took note of specific language patterns and nonverbal cues, such as the interchange between “I” and “we,” as well as the use of fillers like “uhms” and instances of laughter.	
	Conceptual comments	Reviewers identified and described the leadership strategy demonstrated by the participant.	
*Step 3*. Developing emergent themes		The primary reviewer identified the general competencies applied and how they were used in each participant’s unique experience. The secondary reviewers refined this by specifying the intrapersonal and interpersonal competencies demonstrated, along with their impacts.	
*Step 4*. Searching for connections across emergent themes	Abstraction: an analytic process where emerging themes were clustered with other similar themes.	Excel was used to create a visual map of the emergent themes, which helped identify the superordinate themes. In this case, many themes clustered around the contexts of Strategy Adoption, Teamwork Quality, Client Relationship Management, Ethical Decision-Making, and Industry Adaptation.	
*Step 5*. Moving to the next case	Steps 1 to 4 were repeated individually for each participant.		
*Step 6.* Looking for patterns across cases	a. Identifying paths of meaning: where participants had similar experiences but interpreted them differently, or shared similar meanings despite differing experiences.	The secondary reviewers identified patterns across participant narratives: All participants experienced the five key areas, but interpreted them in different ways. Some had similar experiences but used different strategies. Others applied the same strategy in different contexts, based on their interpretations.	Larkin et al. (2019)
	b. Cross-case thematization	The expert reviewer examined the Excel files containing the audit trail and visual mapping, and analyzed patterns and themes across all participant cases and identified overarching insights.	
	c. Subsumption: an analytic process where an emergent theme becomes a superordinate theme.	The expert identified the overarching interpersonal and intrapersonal competencies that emerged across all participants and the five key areas.	Smith et al. (2009)

The first analysis of the raw qualitative data involved collaboration between the primary reviewer/interviewer and the secondary reviewers. The process began with data familiarization, where the primary reviewer independently transcribed the interviews and performed an independent analysis, focusing on the specific competencies each participant demonstrated in across five key areas of leadership experience, The transcripts and audit trail were then shared with the secondary reviewers, who reviewed the primary reviewer/interviewer’s analysis and carried out their own independent evaluation of the data.

Each reviewer conducted a line-by-line analysis of participant responses, focusing on descriptive, linguistic, and conceptual elements, as seen in Table 3. Particular attention was given to each participant’s unique experiences and the competencies demonstrated in persuading subordinates, fostering team collaboration, managing client relationships, making ethical decisions, and adapting to industry changes. This individual-level analysis captured the nuances of how each participant exhibited intrapersonal and interpersonal competencies, focusing on how each participant uniquely approached these leadership challenges.

**Table 3:** Sample IPA coding process.

Transcript	Descriptive	Linguistic	Conceptual/Interpretative
**P2:** Sometimes, people even stop talking to each other in the office. So it becomes quite difficult. But the good thing is, we are all old enough—we are mature enough to know better. **Interviewer:** Right.	Acting as a mediator when two employees are in a conflict. Asking for suggestions from both sides to determine how to move forward.	Deliberate choice of words in using “you” to emphasize that the subordinate caused the problem yet using “our” to emphasize that both the participant and their subordinate were accountable in resolving the situation.	***‘What is our problem?’, What do you want to happen?’*** Demonstrates self-awareness and perspective-taking—choosing to frame the issue as “our” rather than “your” problem. This reflects an internal value of fairness and relational insight.
**P2:** It’s part of being a worker or an employee. **So I talked to the other person and asked, “What is our problem?,” “What do you want to happen?,” “What are your suggestions?”** Because that’s how I handle things when I see a problem. I ask, “What’s your suggestion?” I don’t start by saying, “This is what you should do.” *You gave me a problem—give me a solution. [laughs]* **“You caused this problem, so help me resolve it. But let me hear you first.”**		Participant was laughing as they recounted the memory of asking the subordinate to provide a solution to address the problem.	Reflects a deliberate effort to uncover underlying motivations and unmet expectations, showing cognitive empathy and foresight. The leader is not assuming the solution but seeking clarity from the individual’s perspective. ***“‘What are your suggestions?”*** The leader recognizes that the best solutions often come from those closest to the problem. This shows humility and an internally driven belief in collaboration. openly discussing problems and inviting input, rather than imposing solutions. It reflects a leader who has both the awareness and the interpersonal tact to find collaborative ways forward. The transcript shows self-driven concern for team dynamics, rather than waiting for conflict to escalate. This proactive step indicates the leader has noticed something is “off” and feels a personal responsibility to address it—a hallmark of **perceptiveness.** **Resilience in Personal Challenges (Intrapersonal Competency)**
			***“You caused this problem, so help me resolve it.”*** cooperative and inclusive approach to resolving disputes—not blaming and punishing, but involving the individual in creating a solution. empowering others to take part in the solution. ***“But let me hear you first.”*** This signals a deliberate pause, a conscious decision to suspend judgment and immediate action. It conveys patience and a commitment to gathering all information. Listening first allows the leader to step into the other person’s shoes, to understand their feelings, frustrations, or logic from their point of view. In this way empathy is vital for finding common ground and solutions that address the root causes of the conflict. The transcript implies effective **conflict resolution**, whether formal mediation always starts with understanding *what* the problem is and *who/what* contributed to it. **Resolving Conflicts Through Empathy (Interpersonal Competency)**

Bold text in the transcript and in the Conceptual/Interpretative tab highlights the source of the analyzed excerpts. Terms like “perceptiveness” and “conflict resolution” indicate themes from the first analysis, while “Resilience in Personal Challenges” (Intrapersonal Competency) and “Resolving Conflict Through Empathy” (Interpersonal Competency) reflect themes from the second analysis.

Subsequently, a member checking process was conducted. The primary reviewer/interviewer facilitated this step to maintain consistency in interactions among all participants. Participants were encouraged to correct or clarify the researchers’ interpretations, and any adjustments were noted in an Excel tracking table to reflect revised understandings. This helped deepen the researchers’ insight into each participant’s context and experience.

To further reduce bias and ensure analytical validity, an FGD among all the researchers was held to evaluate the outcomes of both the first analysis and feedback from the member checking.

The second analysis followed, in which an expert reviewer evaluated the themes, and identified recurring patterns across participants’ experiences in the five key leadership areas. A cross-case thematization followed, comparing similarities and differences in participants’ experiences to refine the prominent intrapersonal and interpersonal themes further. This process resulted in the identification of five core intrapersonal and five core interpersonal themes. Importantly, competencies exhibited by even a single participant were not excluded; thus, diversity in expression was preserved. This acknowledges that while patterns existed, each participant enacted competencies in contextually unique ways. A second FGD was subsequently conducted to review and discuss the final themes, ensuring alignment and credibility.

Overall, the analytic process moved from an idiographic focus on individual lived experiences, such as how each participant persuaded subordinates or adapted to industry shifts, to a group-level synthesis that uncovered convergences and divergences in leadership approaches across different contexts of the participants. This outward analytic movement enabled a nuanced understanding of how interpersonal and intrapersonal competencies intersect in practice, ultimately providing a rich, interpretive account that honors both the multiplicity and shared meaning within participants’ lived experiences.

## 4 Results

The researchers initially conducted an analysis to gather insights from the five participants on strategies they would use to effectively address or prevent common challenges within their departments. This analysis focused on five key areas: strategy adoption, teamwork quality, client relationship management, ethical decision-making, and industry adaptation. The subsequent sections explore the themes identified in each key area.

### 4.1 Strategy adoption

Participants reflected on how they led their teams in the process of Strategy Adoption, revealing a range of leadership competencies that helped minimize resistance to change. Each leader demonstrated distinct approaches, shaped by their personal leadership styles and the specific contexts in which they operated. P2 adopted a firm, directive approach, particularly in the face of resistance. “You have to accept that this is what we will use… I deleted the Excel so they would not revert to it,” they explained, demonstrating a non-negotiable stance that reinforced commitment to the new system. Their leadership relied on structure, boundary-setting, and decisive enforcement to guide the team through transition. In contrast, P1 influenced change through trust rather than authority: “I’m not a pushover. They will do it not because I’m a manager, but because I feel like they trust me.” Grounded in relational credibility, P1 fostered voluntary alignment by building strong interpersonal connections. While both leaders were intentional in setting expectations, P2 emphasized structural clarity, whereas P1 drew strength from interpersonal trust. Despite their differing styles, both approaches ultimately led to effective Conflict Resolution and Negotiation during strategy implementation.

P3 highlighted the importance of participative decision-making: “I relay the information to my team because I need their buy-in. Once they say ‘yes,’ it means we are accepting the responsibility.” Similarly, P4 stressed the role of trust in securing team alignment, noting, “One of the best practices to get their buy-in is you have to build their trust.” While both prioritized engagements, their methods diverged—P3 fostered alignment through shared decisions, while P4 emphasized long-term commitment through relational trust. Together, their strategies reinforced the importance of Building and Strengthening Relationships in supporting smoother strategy adoption.

Further illustrating relational leadership, P2 resisted relying solely on positional authority: “You’re not power-tripping just because you’re the boss. You need to understand the person.” This revealed a desire to lead through empathy and emotional intelligence. Instead of imposing directives, P2 adapted to individual circumstances to guide adoption. P4 echoed this sentiment, stating, “The authority shouldn’t be imposed [immediately]. It should be the target performance and objectives that should be imposed.” Their method focused on outcome clarity and shared purpose rather than power. While P2 led through empathy and P4 through purpose-driven alignment, both rejected authoritarian tactics—demonstrating Ethical Integrity and Moral Courage in guiding their teams through complex change.

P2 also described using calm, measured communication to handle resistance, linking short-term decisions to long-term impact: “You need to talk to them calmly… show that this is the result if we don’t act.” This approach reflected a deeper belief in reasoning as a motivational tool. In contrast, P3 emphasized ethical leadership developed through self-awareness: “There has been a self-reflection, and I have become more transparent [about the context of a decision].” Rather than assert authority, P3 relied on openness to build trust. In their own ways, both leaders nurtured psychological safety—P2 through calm, forward-looking dialogue, and P3 through vulnerability and introspection—demonstrating how ethical leadership supports Fostering an Ethical Culture.

Leaders also focused on proactive engagement to prevent conflict. P3 emphasized relationship-building: “I connected with them… it’s easier for us to work together if we know each other,” showing how familiarity facilitated smoother collaboration. P5, meanwhile, encouraged participation from the outset: “We consolidate all the ideas, and we present them with the team… we pitch in and we get their insights.” By structuring early collaboration, they fostered shared ownership. While P3 emphasized one-on-one rapport and P5 leaned on collective input, both exemplified how inclusive practices support Proactive Conflict Prevention.

Building on these relational efforts, leaders reflected on how they navigated complex decisions. P3 recounted how their team adapted during the pandemic—“one of the external changes that we did not have control of but we had to manage”—showing a capacity to recalibrate under pressure. In contrast, P5 emphasized the need for stakeholder buy-in: “You don’t initiate any big change because you don’t have buy-in.” Their method involved documenting current processes and streamlining only where needed to minimize disruption. P3 prioritized agility and forward planning, while P5 focused on inclusivity and operational continuity. Despite differing strategies, both underscored the need to align decisions with team realities—demonstrating how leaders develop Strategic and Inclusive Decision-making through a blend of adaptability, collaboration, and contextual sensitivity.

P1 also shared how transparency strengthened their leadership: “Our communication is very open. I can tell them what I’m thinking, and they can also suggest,” describing how mutual exchange encouraged participation. P4 took a complementary approach, emphasizing affirmation: “You always have to make them feel that their suggestion is always valued, that their suggestion is not disregarded.” For P4, psychological safety rested on emotional validation. While P1 emphasized dialogic exchange and P4 prioritized recognition, both fostered trust through openness—each contributing to Creating a Psychologically Safe Environment that encouraged team engagement.

When navigating the discomfort of change, leaders also relied on reassurance and clarity. P2 offered empathy: “It’s just difficult for now. Once you have had it or you get used to it… it’s going to be very easy for us,” helping normalize resistance while setting positive expectations. P3 provided cognitive structure: “I explain what’s the reason, the plan there, and the context of why it has been assigned to our team.” Their clarity helped teams see their role in a broader strategy. While P2 focused on emotional support and P3 on logical explanation, both demonstrated Effective Communication in Difficult Situations to sustain engagement through change.

Rather than treating strategy adoption as a transactional process, participants described it as a dynamic practice of leading through complexity. Across their accounts, effective leadership involves deliberate, often personal choices—whether enforcing structure, building trust, anticipating conflict, or guiding change with moral clarity. These competencies were not just enacted but cultivated through lived experience and reflection. Leaders showed that fostering openness, collaboration, and integrity not only minimized resistance but also built organizational resilience. Their narratives suggest that strategic success lies not in controlling outcomes, but in cultivating environments where people can confidently and ethically contribute to change.

### 4.2 Teamwork quality

Participants shared examples of how they improved teamwork by handling internal problems. Each person had their own view of what good teamwork means, based on their job and experience.

P1 emphasized that teamwork was strengthened through shared struggles during high-pressure periods, such as bidding and tight deadlines. These intense situations fostered unity and reinforced a strong sense of collective responsibility. P1 recalled, “We had to work overnight. She did not leave me then. We worked overnight until 7:00 a.m. We finished the bid,” illustrating how mutual effort deepened team commitment. Now serving as a supervisor, P1 applies this experience by modeling Supportive Leadership—choosing to work alongside their team rather than simply delegating. “So I saw from my director, so that’s what I’m doing with my team,” they explained, promoting a hands-on, team-centered leadership culture.

P1 emphasized that a leader’s tone and presence significantly impact team dynamics. “Don’t be overbearing… Being authoritative discourages collaboration,” they explained, highlighting the importance of a balanced, approachable leadership style. While P1 did not experience authoritarian leadership, they acknowledged its potential to hinder open communication. In contrast, Democratic Leadership fosters idea-sharing and inclusion, enabling diverse perspectives to surface and drive innovation.

P1 also described the value of saluhan—a culture of mutual support. “We have each other’s back… We don’t leave each other hanging,” they said, reflecting their belief in shared responsibility and consistent support. For P1, being a Team Player means stepping up when others struggle and showing reliability throughout the team’s journey. Similarly, P5 shared, “The majority of the people in AS have a ‘C personality’ type. Some may have an ‘I,’ ‘IC,’ or ‘IS’ type, but most are ‘C.’” This underscores that teamwork also involves adapting to the team’s dominant personality and working style, supporting cohesion and collaborative success.

P3’s view of teamwork quality emphasizes building bonds beyond work hours to strengthen cohesion. Acknowledging the nature of their work, P3 shared, “We often organize out-of-town activities on our own dime, and these trips have been instrumental in strengthening our bond.” While social in nature, these gatherings serve a strategic purpose—fostering trust, morale, and group identity, all essential for sustained collaboration.

For P3, shared experiences improve adaptability and motivation within the team. Highlighting the importance of consistent support, they said, “To be flexible, to be present, to be reachable… because motivation is really important in a team.” This availability reflects P3’s Courage—the ability to stay emotionally grounded and responsive even under pressure, ensuring others feel supported. P3’s continued efforts also reflect Resilience, shown not just by enduring challenges but by responding with empathy and dedication. By valuing emotional connection and being consistently present, P3 cultivates an environment where team members feel seen and motivated. This relational resilience strengthens both individual performance and collective success, reinforcing the role of emotional presence in effective teamwork.

While P1, P5, and P3 all value Supportive Leadership and being a Team Player, they differ in approach. P1 focuses on solidarity during high-pressure tasks, while P3 fosters cohesion through voluntary social bonding. P1 is more task-driven; P3, relationship-focused. Despite this, both emphasize leader presence and availability as essential to cultivating Courage and Resilience within the team.

P4 offered a perspective on teamwork focused on managing internal issues through transparent communication. Recalling a case with a non-performing team member, P4 shared, “We distributed the tasks to others who were more efficient… There was probably an internal issue before.” Despite coaching efforts, performance did not improve, prompting a clear decision and open explanation to the team. “The team adopted a transparent approach… explaining the reasons behind the decision and the consequences.”

This Proactive Communication reduced misunderstandings, promoted fairness, and preserved morale. By addressing the issue directly and honestly, P4 demonstrated a Conflict Resolution strategy grounded in clarity and accountability. Their approach highlights how openness can defuse tension and reinforce trust, ensuring alignment and team cohesion during challenging situations.

P2 also addressed conflict through Proactive Communication to maintain team harmony. Serving as a mediator, P2 shared, “I tell them, ‘Just tell me, and I’ll talk to them… That way, we can avoid argument,’” highlighting their role in preventing escalation. This reflects emotional intelligence and empathy, allowing P2 to manage issues without damaging relationships. P2 stressed compassionate communication during tense moments: “You have to be light with them. It still has to be gentle.” For P2, Empathy builds trust and fosters a respectful environment where team members feel heard.

Complementing P4’s approach, P2 emphasized listening before acting. In a conflict, they said, “You caused this problem, so help me resolve it. But let me hear you first,” showing a balance of accountability and understanding. P2 also clarified intentions to avoid misinterpretation: “That’s not my intention to make it difficult… maybe it was just misinterpreted,” demonstrating how reflection and dialogue de-escalate tension and strengthen team cohesion.

P2 demonstrated strong self-awareness and Perceptiveness by recognizing early signs of conflict and addressing them through reflective questioning: “What is our problem? What do you want to happen? What are your suggestions?” This approach created space for shared problem-solving and reinforced a culture of trust and collaboration.

Both P2 and P4 utilized Proactive Communication but with differing styles. P4 focused on structured transparency to uphold fairness and morale, while P2 relied on emotionally attuned mediation grounded in Empathy and Perceptiveness. Their contrasting tones highlight how effective conflict resolution depends on situational context and interpersonal dynamics.

In summary, all participants emphasized the importance of teamwork quality and conflict management, but through diverse approaches. P1 and P3 embodied relational leadership—through task-based solidarity and social bonding—demonstrating Supportive Leadership, Democratic Leadership, Courage, and Resilience. Meanwhile, P2 and P4 addressed conflict through different communication strategies: P2 through empathy and emotional insight; P4 through clarity and accountability. Despite varied methods, each leader reinforced that effective teamwork thrives on presence, emotional intelligence, and a shared commitment to collaboration and trust.

### 4.3 Client relationship management

When participants were asked how leadership competencies contribute to maintaining a strong client relationship, P1 promotes boundaries to manage client expectations, “What I tell the team is, don’t stress yourselves out. Just tell them, ‘Okay, we’ll handle it. We’ll get back to you tomorrow morning at the earliest.’ So they would also know that there are limits.” While P2 demonstrated empathy and politeness in resolving conflict, “Just be polite. My rule of thumb is: treat others the way you want to be treated. If you don’t want to be yelled at, don’t yell. If you want respect, give respect. If you want others to be polite when giving you instructions, be polite when talking to them. Meanwhile, P4 prioritized client needs to ensure client satisfaction, “Establish rapport with the client during the initial meeting when the project is awarded. Make them feel valued right away and prioritize their needs.” Reflecting on clarity, empathy, and proactive engagement, Effective Communication was showcased.

Emphasis on prioritizing rebuilding client trust, P1 shared, “I did not stop following up with our after-sales team, who handled it until it was resolved. It did not take more than two days to resolve the problem.” In addition, P4 highlights patience toward unreasonable demands and timely acknowledgment, avoiding tension “Of course, there are clients who make excessive demands that go beyond the scope of the contract. In those cases, you just have to be kind and patient,” and preventing conflict “Even if you can’t provide an immediate answer, acknowledging their message… is important. The main thing is that they feel heard.” Demonstrating Conflict Resolution, leaders maintained professionalism and fostered positive relationships with clients, even in challenging situations.

Furthermore, P1 highlights proactive responsiveness to resolve issues and maintain rapport. “It’s making them feel that they are a priority, that if they need anything, we will find a way to do it,” In contrast, P4 designed solutions that ensures clients feel valued, “Even if you can’t provide an immediate answer, acknowledging their message… is important. The main thing is that they feel heard.” Fostering Client Engagement, participants emphasize the importance of ensuring client trust and loyalty.

Promptly, P1 ensured satisfaction and regularly engaged with clients, “We’ve been keeping in regular contact with the client, asking questions to ensure their satisfaction.” Furthermore, P5 emphasizes the importance of holding accountability and viewing complaints as opportunities for improvement. “We’re client-facing, so there were a lot of complaints.” The demonstration of leaders seeking Feedback and Improvement from the client strengthens trust and accountability. Statements emphasized that they use feedback as a tool for growth to ensure better client experience and long-term success.

Maintaining composure under pressure, P2 demonstrated excellent customer service by emotional regulation, “Because even if they are already angry, you still think about the positive side…,” While P4 reflects a relational approach and exhibited patience and kindness toward the client, “Of course, there are clients who make excessive demands that go beyond the scope of the contract. In those cases, you just have to be kind and patient.” these insights from participants emphasize that Customer Service Excellence is giving more than meeting basic expectations.

To uphold the team’s wellbeing, P1 set boundaries by recognizing client limitations, “Just tell them, ‘okay, we’ll handle it; we’ll get back to you tomorrow morning at the earliest.” While P2 reflects on past emotional struggles, to build personal resilience and mentor others, “I told them, ‘Don’t worry, they will not hurt you. Just be confident in yourself and know that you have done nothing wrong.’ That’s the advice I needed when I first started this job.” Highlighting the significance of Self-awareness in the client relations context, recognizing personal space and reflecting on learning enables leaders to maintain emotional regulation and foster productive relationships with clients.

Balancing urgency through structured response, P1 exemplified, “I do respond after office hours… I make sure to get back to them within the day with a solution or an answer from the project management team.” In addition, P4 willingly extend effort beyond regular hours to meet client needs, showcasing commitment amid personal sacrifice, “We often go beyond what’s expected of us, even working on Saturdays and during holidays like Holy Week… We usually try to respond, especially if it’s urgent.” As leaders make time for client concerns, they demonstrate Self-management through disciplined efficiency, perseverance, and adaptability.

Demonstrating optimism during high-pressure client interactions, P2 shared, “Because even if they are already angry, you still think about the positive side… ‘How do you do that? Sir (name of executive) is already angry, but we are still calm.” While P5 emphasizes the need for perseverance, “That’s the reality… There were complaints from the clients and complaints internally because we are partners with sales in providing service to the clients.” Showcased Resilience involves maintaining a positive outlook to foster continuous improvement and build lasting connections with clients.

Giving importance to client feedback, P1 shared their practice, “We’ve been keeping in regular contact with the client, asking questions like, ‘How are you? How is our project implementation going? Do you have any feedback?’.” In addition, P3 emphasizes strategic industry awareness, focusing on proactive learning through competitor analysis to maintain market relevance. “We have to remain competitive by doing a competitor analysis. We need to know how our competitors are performing outside. We strategize on pricing and approach.” As leaders pursue knowledge, skills, and self-improvement, they promote Professional Development to innovate solutions and build enduring trust with clients.

Emphasizing the importance of transparency and accountability, P1 ensures trust and integrity, reinforcing the client’s confidence in their professionalism and dedication, “But then we make it a point that we still get in touch with the end-users since we have the initiative to talk to them.” Similarly, Participant 4 demonstrated prioritizing client needs and fostering rapport, “Establish rapport with the client during the initial meeting… Make them feel valued right away and prioritize their needs. If they have scheduling conflicts, be accommodating.” Upholding Ethical Standards, the leaders fostered trust and maintained integrity in client relations.

### 4.4 Ethical decision-making

Participants were asked about how they handled situations when choosing between practical and ethical solutions. Mixed responses were observed as the context played a role in the participants’ experiences.

In the case of P1, they did not have a difficult time choosing ethical solutions even when odds are against them, evident in their statement, “It was as if they were accusing us. I don’t do that, but I would explain to them to let them know what happened to resolved the conflict…” Although they encountered a dispute with another department, P1 opted to explain and clarify what happened instead of retaliating, even if they had the right to do so. It points to Strengthened Interdepartmental Relationship because P1 was able to elaborate on better work boundaries that could prevent future conflicts. Additionally, P1 prioritized resolving the conflict, demonstrating their Negotiation Skills in a tense situation. These highlight how P1 responds ethically, as they ensure that each department’s needs are satisfied.

For instance, P1 emphasized that they do not have difficulty choosing between what’s ethical and practical. As stated, “I do not find it challenging when choosing what’s right because the organization itself demands integrity.” Along with that, P1 mentioned, “You should have integrity in every transaction, whether internal or external…” This showcases that, regardless of who they are dealing with, they aim to uphold their ethics and follow the proper process. This demonstrates the department’s Strong Ethical Culture, as it leads to a uniform approach to their work. Furthermore, it elicits Moral Courage, given that the level of authority does not affect their process. Thus, regardless of the client or organization, P1 encourages its subordinates to uphold Ethical Decision-Making.

Despite a strong imposition of ethics on subordinates, P1 values receiving insights from colleagues when it comes to establishing an effective solution. A collective approach is observed in the quote by P1, emphasizing, “You have to weigh things… What solution can be done so that the company can benefit the most through your department?” This encourages feedback because weighing things means considering several perspectives. Moreover, P1 views the department as a group, which implies that Psychological Safety is established, as contributions are viewed through a group lens rather than individually. In relation to that, the statement, “From what I can see, the solution is to mitigate any conflict that may arise from a certain situation,” illustrates that P1 has a solution-oriented approach. This can be observed in how they aim to gather insights from different angles to reach the most efficient method. P1 then displays Conflict Prevention Skills, considering only the short-term but also the long-term.

In comparison, P2’s experiences in handling ethical and practical solutions showed that they had difficulty in addressing dilemmas, as it involved coordinating with employees and top management. Their statement, “I’m an understanding person, so when the top management says to execute it this way, even though it doesn’t align with my principles, I don’t resist…” shows that they would choose to comply instead of raising their concerns. In another setup, P2 shared that, “During the pandemic, when probationary employees had to be laid off, there was no strategic approach on how the company could cope but to let go of new employees.” This shows P2’s capacity to handle Difficult Conversations, given that they are aware of how valuable it is to have a job during the pandemic. As P2 ultimately resorted to employee termination to ensure the company’s survival, they were able to show their Decision-Making skills in a time of uncertainty.

Similarly, P3’s approach to choosing ethical and practical solutions aligned more with P1, where ethics was strictly enforced. The statement by P3, stating that “You can’t do practical if your ethics are going to be compromised,” displays a sense of responsibility when making decisions. Although P3 understood that being practical can be an easier way of managing situations, they chose to delve deeper into how a problem can be ethically addressed by considering all angles, rather than opting for a quick-fix solution.

The same approach was exhibited by P4 when managing ethical decisions. As stated, “I think we have not encountered a situation yet where we have to choose the practical side over the ethical one since we are consultants… Maybe I’d be more practical when handling other matters, such as terminating an employee.” This only shows that ethics is non-negotiable for P4 as they emphasized that there is almost no situation where they would consider practicality over ethics. On the other hand, employee termination became an option if it would ensure that everyone would deliver quality work. P4 demonstrates that they consistently uphold Integrity and Conflict Prevention Skills within their department. This was also evident in the way P4 handles data, as observed in their statement, “All our work, including data management, when one respondent’s data is missing, we can’t just say that okay let us fill it up.” Thus, this demonstrates that P4 implements a Strong Ethical Culture that enables them to deliver accurate and high-quality output.

On the other hand, P5 showed the same sentiment as P2 when choosing between a practical and ethical solution. In their statement, “Actually, for this, whenever I’m forced to choose between what’s practical and what’s ethical, I feel that I always put forth what is beneficial for the company,” it’s evident that they perceive it as a dilemma, opting to prioritize the organization. This means that their Decision-Making is highly affected by external factors, considering that there is room for choosing practicality over ethics. In P5’s defense, they said, “It is very easy to say that this is the more ethical choice if you do not know how big of an effect the choice would be if you take a look at it for 5 to 10 years from now.” This scenario highlighted Proactiveness in P5 as they consider how decisions can impact the company even after a few years.

### 4.5 Industry adaptation

Participants reflected on how they adapted to shifts in their industries, revealing varied interpretations of “changing demands” and context-specific adaptation strategies.

For P1, shifting industry demands were defined by the 2020 lockdown, which rendered their paper-based workflows unworkable. Recalling, “We’d work overnight documenting, filing, and compiling everything for paper submission,” P1 highlighted the scale of change. Their use of “we” emphasized Enhanced Collaboration and Innovation, as the team regularly met to reimagine their processes. By stating “What are we going to do?” P1 demonstrated awareness of the urgent need for change and a collaborative approach to addressing it, which are both fundamental components of effective Change Management.

P1 introduced innovations like online submissions, direct agency coordination, and targeted client communication—demonstrating Change Leadership through clear communication and strategy. Involving the team in service adaptation fostered Employee Empowerment, enhancing confidence, motivation, and resilience.

P1 further emphasized the role of Learning Agility in sustaining organizational performance amid adversity: “You would find ways to innovate so you can still serve your clients.” This willingness to seek new knowledge and adopt novel approaches reflected their capacity to remain competent and competitive in a rapidly changing environment. Additionally, their assertion, “It’s a bit difficult to do, but if you really want it, when you aim for something, it becomes a motivation to achieve your goals and objectives,” illustrated the role of perseverance and goal orientation in fostering creativity and resilience under pressure.

While P1’s context was centered on the pandemic, P4 focused on shifting client demands. In response to a newly introduced client guideline that rendered their existing service obsolete, P4 took proactive measures to reposition their organization by organizing a seminar to reframe their value proposition: “We invited all our government clients to the seminar to position ourselves at the forefront and establish this organization as the premier third-party service provider for this new guideline.” This action demonstrated anticipatory problem-solving and strategic foresight, hallmarks of agile leadership in dynamic markets.

Despite differing contexts and approaches, P1 and P4 both demonstrated More Robust Innovation and Problem Solving in navigating complex challenges. Their proactive, adaptive responses embodied Change Leadership, fostering motivation and a supportive environment for growth and resilience.

P2 viewed changing industry demands through the lens of technological adaptation, emphasizing the need to support their team in learning new digital tools. They highlighted the role of Communication Skills in facilitating this transition. When introducing new software that enhanced work efficiency, they stated: “Be very gentle, especially when introducing something new. Because, you know, they are not used to that.” P2 emphasized empathy and patience to ease their subordinates’ frustrations, creating a psychologically safe environment that reduced resistance and enabled smoother adoption of new systems.

P2 also embodied Openness to New Ideas by discarding outdated methods in favor of evolving technologies. Reflecting on their own learning journey, they shared, “At first, I was also hesitant. But I did not have a choice. Things are evolving, so we need to keep up,” illustrating a shift from reluctance to acceptance in response to changing industries.

Similarly, P4 demonstrated openness to technological change, initially expressing skepticism about the use of Artificial Intelligence (AI): “I felt it was unfair because I was relying on AI and asking it for help. But now, I recognize its value.” Both participants’ willingness to embrace technological innovations enabled them to drive progress and enhance productivity through improved workflows.

In contrast, P5 demonstrated openness to new ideas in a broader strategic context by responding to client feedback during the pandemic: “We have more clients because of the pandemic and everything shifted to digital. Information security received more attention, and we took an information security certification…” In doing so, they used client feedback to guide service enhancements.

P5 also demonstrated openness to new ideas by regularly reviewing client memos to assess whether their current assessments were aligned. They reflected, “We take a look at the memo and see… Will our product address this memo or not. What can we do to adapt it, or to change or to update? What are the possible improvements that can be made to the products?” This shows their willingness to consider external input and incorporate new perspectives to improve their services. Furthermore, P5 recognized the strategic value of informal knowledge networks, explaining: “Don’t confine yourself to formal channels because those formal channels only provide limited information. Listen to what others are saying, even the gossip.” This insight emphasized the importance of tapping into diverse information sources to anticipate emerging market trends.

P3 echoed this perspective, highlighting the centrality of networking in securing clients and generating new opportunities: “Building a network is essential because without it, you can’t prospect. To prospect, you must expand your network.” This orientation toward relationship-building supported strategic adaptability and business sustainability in rapidly evolving environments.

Thus, while P2 and P4 showcased openness to technological innovation to streamline internal operations, P3 and P5 showed how openness to client input and network insights informed service design and sustained market relevance.

Beyond digital transformation, P2 also interpreted industry change in terms of navigating flexible work arrangements. They shared the importance of maintaining strong employee relationships in hybrid work settings: “It helps them realize they have officemates aside from the ones they sit next to at home or in their department.” This practice of fostering informal support networks helped reduce isolation and promote cohesion, which is critical to Team Adaptability in hybrid environments.

Lastly, P3, like P1, framed their experience of changing industry demands around the pandemic. ‘It’s a year of challenge for all of us,’ they noted, acknowledging the widespread disruptions brought about by the pandemic. However, unlike P1, P3 emphasized a different core competency, Resilience. “That you are resilient, you’re hopeful that everything will be okay again.” This sense of optimism allowed them and their team to maintain momentum and persevere through adversity, a mindset essential for navigating prolonged periods of uncertainty.

### 4.6 Second analysis

**Figure 2:**
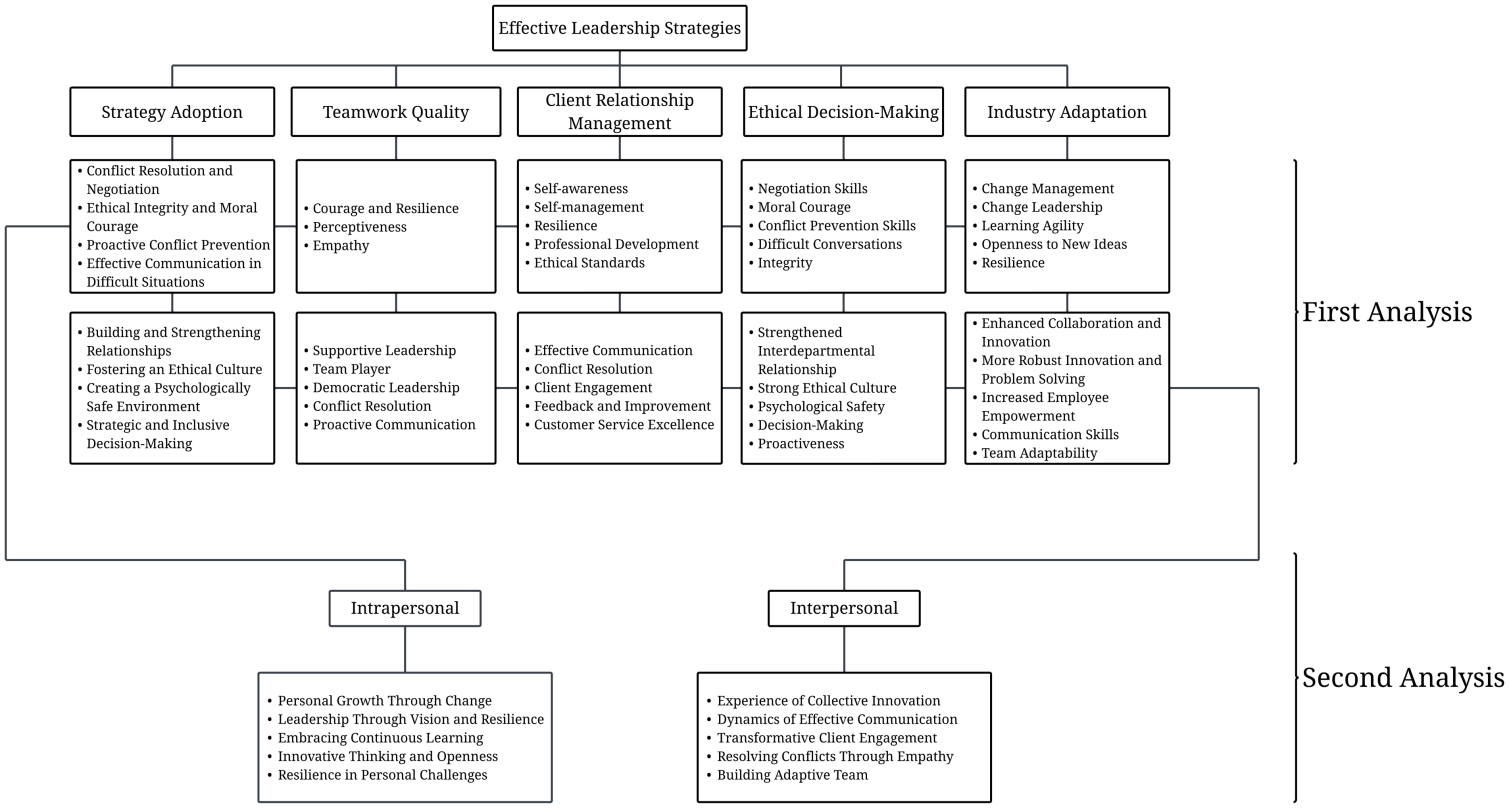
Effective leadership strategies.

The first analysis identified core leadership competencies common to all participants, while the second analysis examined how these competencies were applied in specific contexts, revealing variations in their use. This nuance offered a clearer understanding of their adaptability across situations. The study’s iterative analysis provided a comprehensive view, as illustrated in Figure 2.

Interpersonal competencies are skills and behaviors that facilitate effective interactions and relationships, which are essential for fostering strong bonds, promoting an ethical culture, and cultivating a safe and inclusive space for decision-making.

The first interpersonal competency derived is Experiences of Collective Innovation. It represents the collaborative efforts that resulted in innovations as participants provided diverse ideas to facilitate creative problem-solving. For instance, this covers leaders’ efforts to engage with various technologies by maximizing their use to improve team productivity.

In relation to that, the second interpersonal competency that emerged was the Dynamics of Effective Communication. This represents leaders’ capacity to actively listen to their subordinates while practicing clear and respectful dialogue. With this competency, trust and understanding becomes established, making it easier to manage client expectations while maintaining smooth working relationships. Other than that, giving and receiving feedback was a practice that contributed to the dynamics of effective communication.

The third interpersonal competency that emerged from five different sub-themes is Transformative Client Engagement. This was identified as a recurring pattern among leaders as they showed responsiveness and individualized exchanges to clients. Such became key in nurturing mutual respect and understanding, more so as sincere interactions improved client satisfaction and retention.

Meanwhile, the fourth interpersonal competency generated is Resolving Conflicts Through Empathy. Leaders highlighted here how they utilized de-escalation techniques to manage tense situations. Because of the ability to mitigate arguments, these did not escalate as a bigger issue. Through this, positive relationships were maintained despite the disagreements they encountered.

Lastly, the final interpersonal competency that was recurring among leaders was Building Adaptive Teams. Navigating challenges as a team was emphasized here as the competency represented leaders’ ability to guide their teams during challenges. Teams were developed to be flexible and resilient as leaders assisted them to adjust to new conditions while maintaining morale and productivity. As the capacity to adapt was observed on the whole team, it became a factor that further unified them. Thus, it then implies that participants are leaders who were able to navigate the changing landscapes of the market while giving direction to their subordinates.

Intrapersonal competencies enable leaders to develop self-awareness, emotional regulation, and adaptability. Their inner world is navigated by fostering resilience, purpose, and a growth mindset. Developing intrapersonal competencies of leaders enhances overall leadership effectiveness.

Firstly, Personal Growth Through Change emphasizes the transformative power of adapting to new circumstances, challenges, or environments. Personal growth through change highlights leaders’ resilience, self-reflection, and planning in adapting to challenges. Intrapersonal skills like self-awareness and patience help navigate transitions, fostering strength and adaptability in dynamic environments.

In addition, Leadership Through Vision and Resilience focuses on the importance of a leader’s ability to inspire and guide their team with unwavering commitment during times of transformation. Leadership through vision and resilience highlights leaders’ ability to inspire teams, navigate challenges with adaptability, and foster unity through inclusive decision-making, clear communication, and a steadfast commitment to strategic goals.

Moreover, Embracing Continuous Learning highlights the importance of maintaining an open mindset and continuously seeking knowledge to stay agile in a constantly evolving landscape. Embracing continuous learning emphasizes leaders’ commitment to self-improvement, adaptability, and innovation. By seeking new knowledge, they inspire teams, foster agility, and strengthen resilience, driving personal and organizational growth in dynamic environments.

Furthermore, Innovative Thinking and Openness underscores the significance of staying receptive to unconventional approaches and fostering a culture of creativity and collaboration. Innovative thinking and openness highlight leaders’ openness to new ideas and unconventional approaches, fostering innovation, adaptability, and collaboration. This mindset enhances problem-solving, inspires teams, and builds resilient, forward-thinking organizational cultures.

Finally, Resilience in Personal Challenges refers to the ability to remain steadfast and optimistic, overcoming adversity while pursuing long-term goals. Resilience in personal challenges refers to the ability to remain steadfast and optimistic in adversity, allowing leaders to continue pursuing their goals despite setbacks. Leaders demonstrated resilience and optimism, managing challenges with clarity, mindfulness, and goal-setting. Their perseverance inspired teams, fostering a culture of adaptability, focus, and progress in overcoming obstacles and driving organizational success.

## 5 Discussion

Effective leadership competencies contribute to individual and organizational success by inspiring, guiding, and influencing others to achieve common goals (Chow and Singh, 2023). The study conducted an in-depth analysis of leaders’ experiences using IPA, identifying several key competencies in strategy adoption, teamwork quality, customer relationship management, ethical decision-making, and industry adaptation. After reanalyzing emerging themes among all key areas, it was revealed how interpersonal and intrapersonal competencies influence leadership, bridging the gap between theory and practice and highlighting the importance of holistic leadership in achieving organizational success.

### 5.1 Interpersonal competencies

The first interpersonal competency theme reveals Experiences of Collective Innovation cultivated by teamwork and shared commitment. P1 illustrated this dedication: “We’d work overnight documenting, filing, and compiling everything for paper submission.” This collaborative approach enhances innovation through improved coordination, idea-sharing, and systematic planning. This contradicts the findings of Heldal (2023) to some extent where the role of Design Thinking (DT) to innovation is emphasized. In this case, divergent and convergent thinking is prioritized along with authority and discipline rather than merely focusing on collaboration.

Meanwhile, P1 noted: “It’s a bit difficult to do, but if you really want it—when you aim for something—it becomes a motivation to achieve your goals.” Leaders foster environments encouraging creative thinking and experimentation. By leveraging technology and embracing team-oriented strategies, organizations generate innovative solutions while building foundations for adaptive decision-making, sustaining agility amid evolving industry demands. Still, Barroga et al. (2021) showed how micro, small, and medium businesses in the Philippines varied in how they adopted smart manufacturing (SM) technologies as technological knowledge and implementation played a role. Thus, the results add to the existing knowledge that for collective innovation to transpire, collaboration, technological changes, and team-oriented strategies are important.

The second theme reveals Dynamics of Effective Communication where leaders actively encourage team dialogue while serving as bridges for understanding and resolution. P4 emphasized collaborative decision-making: “I relay the information to my team because I need their buy-in. Once they say ‘yes,’ it means we are accepting the responsibility.” This builds shared accountability through open communication, mutual trust, and consensus-building. This approach contradicts to Mapoy et al. (2021) which revealed that many Filipino organizations still depend on top-down decision-making that limits employee involvement and reduces team agility.

While P2 highlighted the balance between directness and empathy: “You need to talk to them calmly… show that this is the result if we don’t act.” P4 treats communication as both a tool for guidance and caring for team members while maintaining organizational responsibility.

This study addresses the gap in leadership accountability and employee wellbeing identified by Angeles (2024). The theme, drawn from the insights of P2 and P4, illustrates how leaders leverage communication to promote care and shared responsibility. This reflects a shift toward collaborative leadership that balances people and performance, where communication fosters shared leadership, compassion, and organizational accountability.

The third theme is Transformative Client Engagement as a defining leadership practice, where leaders exceed expectations by prioritizing client satisfaction beyond standard requirements. This theme goes beyond immediate problem-solving to ensure clients feel valued and heard. As P4 noted, “Even without an immediate solution, acknowledging their message matters—what’s key is making them feel heard.” Moreover, exceptional leaders also demonstrate conflict resolution by taking personal ownership of issues until resolution. P1 exemplified this: “I persistently followed up with our after-sales team, ensuring the problem was resolved within two days.”

The findings address gaps in both Tabrani et al. (2018) and Namasivayam et al. (2014). While Tabrani focused on emotional factors like trust and intimacy, and Namasivayam emphasized indirect leadership through employee empowerment, both overlooked direct leader-client interaction. The theme fills this gap by showing how leaders build satisfaction and loyalty through personal responsiveness, empathy, and active problem-solving.

In summation, the theme fosters loyalty by combining conflict resolution, forging strategic, long-term partnerships grounded in professional integrity. By consistently addressing tensions and upholding transparency, leaders solidify trust and mutual benefit, distinguishing their organizations in competitive landscapes.

Results showed that Resolving Conflicts Through Empathy was a common theme among participants as they managed disputes with patience and understanding. Findings showed that participants were equipped with conflict resolution while maintaining accountability among colleagues. This was evident in P4’s passage, “You caused this problem, so help me resolve it. But let me hear you first,” where the leader identified the person accountable for the problem and gave them an opportunity to be heard, rather than punishing them. Also evident in P1’s passage, “I would explain to them to let them know what happened to resolved the conflict…” is the aim of managing disputes through clarification.

While empathy was key to resolving conflicts in the study, Ilac and Presbitero's (2022) research revealed that Batad’s indigenous leaders employ several conflict management processes. They described conflict management as complex and having different levels, which requires both humility and unbiasedness from the leaders. This illustrates the distinction between how indigenous leaders manage conflicts and the conflict management strategies employed by corporate leaders. The study then shows how empathy can be more pronounced in leaders in a private organization.

Building Adaptive Teams was the last interpersonal theme to emerge among the participants. Results illustrated that participants adopted a supportive approach and acted as team players, evident in P1’s statement, “We have each other’s back… We make sure we don’t leave each other hanging,” to create an adaptive team. Participants even spent their own money to strengthen their teams, as observed in P3’s passage, “We often organize out-of-town activities on our dime, and these trips have been instrumental in strengthening our bond.” While it is an informal approach to enhancing team cohesion, interactions outside work played a role in the resilience of team members.

This differs from the study by Isip (2022), which identified value creation, a knowledgeable workforce, and organizational resourcefulness utilizing technology as key factors in the adaptive capability of micro agribusiness firms in the Philippines. In this case, adaptability was based on the company’s products and services. Furthermore, adaptability was attributed to proactivity in global companies from Western countries and parts of Asia when work performance adaptability was assessed (Villalobos et al., 2020). This implies that the current findings illustrate how supportive leaders who act as team players are another factor to consider when building an adaptive team.

### 5.2 Intrapersonal competencies

Personal Growth Through Change emerged as the first theme. Leaders embraced discomfort with growth mindset. P2 noted, “Once you have had it… it’s going to be very easy for us,” showing openness to learning amidst challenges, contrasting with Carbonell-Laguio’s (2023) findings on destructive leadership in Philippine workplace setting, where high power distance and low trust fueled fear-based compliance. In contrast, the leaders in this study leaned into transparency and collaboration, showing that trust and empathy can enable effective leadership even within hierarchical cultures like the Philippines.

Growth mindset was also translated into concrete action. When confronted with operational uncertainty, P1 led their team to co-create solutions under pressure—“working overnight documenting, filing, and compiling everything”—shifting from reactive coping to proactive problem-solving. Similarly, P4 described leading a product seminar to meet changing client needs and “position [themselves] as at the forefront.” This proactive, team-based response reflects kapwa—a Filipino value of shared identity. As Rivera and Ng (2018) explains, Filipino leaders often empower others by fostering a sense of mutual purpose. P4’s use of “ourselves” emphasizes that leadership was exercised in collaboration with their team, demonstrating that personal growth is inherently relational and responsive to external pressures.

Leadership Through Vision and Resilience emerged as second theme. Leaders decide based on ethical conviction and proactive care over authority. As P2 shared, “You have to accept,” while removing fallback options like “I deleted the Excel,” demonstrating commitment to change despite discomfort. This reflects paninindigan—a principled moral stance—which Melendres (2024) describes as the anchor that fastens Filipino virtues to katotohanan (truth). Leaders modeled resolve not by asserting rank, but by practicing consistency and ethical accountability.

Leaders also emphasized values-driven leadership, extending Kim’s (2022) concept of relational leadership in Asian settings. They sustained morale through empathy—leveraging connection as a strategic tool for motivation and alignment. Furthermore, Leaders often invested in early relationship-building to preempt resistance and promote cohesion. This proactive stance extends Wong et al. (2020), positioning trust not as a byproduct but as a foundation of leadership during disruption.

Resilience was further reflected in leaders’ emotional availability. As P3 described, they remained “flexible, present, and reachable,” demonstrating leadership as an active presence, not just structural authority. This aligns with Siason and Siason (2023), whose study of institutional leaders in Philippine higher education highlights flexible leadership grounded in credibility, open communication, and commitment to shared values. In this study, empathy served not only relational ends but empowered teams to move forward with clarity—showing that Filipino leadership sustains vision through moral conviction, foresight, and lived resilience.

Embracing Continuous Learning emerged as a third common theme among organizational leaders in the study, showing its critical role in effective leadership. P1’s drive to “find ways to innovate” during the pandemic highlights how adaptability and a learning mindset can sustain leadership effectiveness under pressure. This insight expands Philippine research on adaptive leadership, which has primarily focused on education (Francisco and Nuqui, 2020) and government sectors (Carada et al., 2023), by offering perspectives from the organizational setting. It underscores the importance of continuous learning—through innovation and adaptation—in navigating volatile environments.

Complementing this, P5 emphasized the value of “doing a competitor analysis” to track trends and identify best practices, enabling them to “strategize on pricing and approach” with strategic foresight and a sustained focus on growth. This reinforces and extends the work of Keya and Kinyua (2025), providing individual-level evidence that continuous learning fuels both adaptive and transformative capabilities. P5’s behavior further illustrates how personal learning practices form the microfoundations of organizational agility and innovation.

Together, these findings respond to Atanassova et al.’s (2025) call to examine middle managers’ roles in organizational learning, demonstrating that a leader’s personal commitment to learning is key to fostering a culture of learning within their teams.

Innovative Thinking and Openness emerged as a fourth significant theme in the study as key drivers of breakthroughs. P4’s emphasis on responsible AI use—“just don’t rely on them for everything”—illustrates how openness paired with critical evaluation can drive both efficiency and innovation. While Romeo and Lacko (2025) found that organizational resistance often hinders AI adoption, this study suggests that such barriers can be addressed through openness, hands-on experimentation, and thoughtful assessment of AI’s practical applications. This insight contributes to the limited literature on AI adoption in Filipino industries, which has largely focused on the government sector (Distor et al., 2021).

Openness extended beyond technology, shaping how participants valued and sourced information. P5 valued informal inputs like “gossip” and client conversations, finding networking at seminars more useful than the content itself. Likewise, P3 attended events to expand networks and stay responsive to client needs—behaviors that supported adaptability and performance. While aligning with Villaluz and Hechanova’s (2019) insights on innovation-supportive leadership, this study adds that informal knowledge and external networks also play a key role. Fostering openness means encouraging leaders and employees to seek insights beyond formal channels, strengthening a collaborative and adaptive innovation climate.

Lastly, Resilience in Personal Challenges emerged as a key theme. This theme contributes to literature by grounding perceptiveness in pakikiramdam, a Filipino cultural value of empathetic attunement (Pe-Pua and Protacio-Marcelino, 2000). Leaders did not perceive disruption as an individual burden, but as a shared challenge—encouraging open dialogue and relational trust. In this way, perceptiveness was not merely observational, but a culturally embedded practice that fostered connection and team resilience.

Furthermore, leaders externalize self-awareness to guide and support others, expanding London et al.’s (2022) description of self-awareness as a largely internal process. As P2 explained, they offered the kind of advice they “needed when [they] first started,” using personal struggles as the foundation for mentoring—an act of resilience that strengthened others through shared reflection.

Leadership accountability is also evident in high-pressure situations. P1 addressed client concerns “within the day,” showing responsiveness and resilience in the face of time-sensitive demands. This promptness fostered trust and transformed a potentially stressful encounter into an opportunity to strengthen relationships. While Abdullah (2023) describes after-sales service as a customer-oriented function, this study reframes it as an act of resilient leadership rooted in personal discipline and accountability.

Throughout the data, leaders exemplified the Filipino concept tibay ng loob—a quiet yet unwavering moral strength (Pe-Pua, 2018), which refers to an inner strength that is stable, courageous, and persistent in the face of adversity. These actions reveal that resilient leadership in the Filipino context is not about bold declarations or overt assertiveness, but about integrity, humility, and deep cultural grounding.

### 5.3 Theoretical and practical implications for organizational leadership

The findings highlight that effective leadership depends on integrating both intrapersonal and interpersonal competencies. This challenges traditional models that treat leadership as either trait-based or relational, calling instead for a holistic approach that recognizes the complementary nature of these domains.

Contemporary frameworks like Goleman’s Emotional Intelligence and Authentic Leadership Theory reflect this integration, linking self-awareness with social awareness and positioning personal growth as a foundation for relational authenticity. The Leader and Leadership Identity Development framework also aligns with this view, showing how leadership evolves from self to collective identity through intrapersonal competence.

These insights refine existing theories. In Transformational Leadership, the ability to inspire is strengthened by traits like resilience. In Leader-Member Exchange (LMX), strong relationships are supported by emotional regulation. Even Katz’s Three-Skill Theory may benefit from recognizing that effective leadership arises from the integration—not separation—of interpersonal, conceptual, and intrapersonal skills.

**Figure 3:**
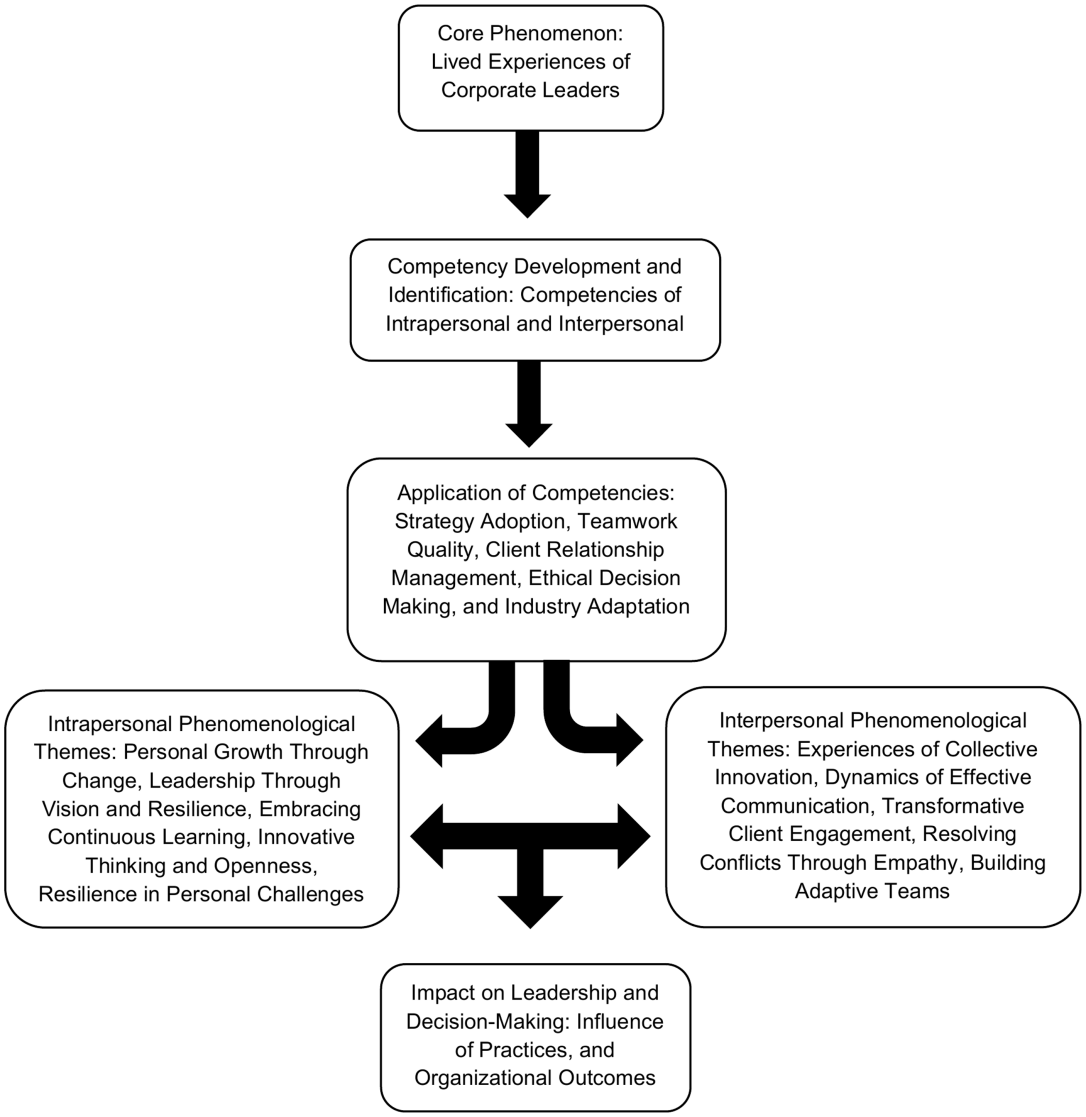
Lived experiences of corporate leaders.

Building on this perspective, the study offers meaningful contributions to the understanding of leadership by emphasizing the dynamic interplay between intrapersonal and interpersonal competencies. Beyond theoretical advancement, the findings yield practical implications for leadership decision-making, organizational practices, and overall effectiveness, as illustrated in Figure 3.

In terms of organizational practices, the identified competencies can inform job descriptions, selection criteria, and performance evaluations. By emphasizing both intrapersonal and interpersonal competencies, organizations can design more targeted leadership development programs, leading to improved coaching, succession planning, and talent retention. These competencies can also be embedded in behavioral interviews and onboarding programs, while leadership assessments can be redesigned to capture the integration of self-leadership and relational skills.

In terms of organizational outcomes, fostering a leadership-supportive culture that recognizes and rewards behaviors aligned with these dual competencies reinforces their importance and promotes consistent demonstration across all levels. As the findings suggest, these competencies often produce a ripple effect—leaders with strong intrapersonal skills build stronger interpersonal relationships, modeling behaviors that contribute to a positive work environment and enhanced employee performance. This collective dynamic ultimately strengthens organizational systems, making them more adaptable, resilient, and better equipped to meet evolving demands.

In summary, the findings integrate empirical insight with practical application, presenting a competency-based framework that advances theoretical discourse on leadership while informing evidence-based organizational decision-making, practices, and outcomes.

## 6 Recommendations

Research limitations were identified, presenting opportunities for further study and prompting future research recommendations.

Firstly, expanding the participant pool enhances generalizability. While this study focused exclusively on five Filipino corporate leaders, a larger, more diverse sample and varying leadership tenures provide broader insights, revealing how leadership competencies evolve and change over time.

Secondly, conducting longitudinal studies adds a temporal dimension to leadership research, revealing evolving competencies and informing development programs beyond the single-point experiences captured in this study.

Finally, cross cultural comparisons can showcase how cultural norms and values influence leadership competencies beyond the Filipino corporate context explored in this study.

Similarly, generational gaps in leadership need exploration to reveal cross-cultural differences and bridge integrational collaboration within organizations.

Indeed, by implementing these recommendations, future researchers can enhance leadership frameworks to address evolving organizational challenges.

## Data Availability

The original contributions presented in the study are included in the article/Supplementary material, further inquiries can be directed to the corresponding author.
