# Supplementary material for: Interpersonal and intrapersonal leadership competencies: an interpretative phenomenological analysis on effective leadership strategies
Source: Front Psychol. 2025 Aug 20;16:1553620. doi: 10.3389/fpsyg.2025.1553620 (PMC12405269; doi:10.3389/fpsyg.2025.1553620)
Supplement: Supplementary file 1 [file Supplementary_file_1.docx]

**Appendix**

Informed Consent Form

**Dear Sir/Madam:**

The Research and Development team at People Dynamics, Inc. is currently developing a new assessment tool. As part of this endeavor, we request your participation in an interview. Below, we outline the terms participants should be aware of and the informed consent form they must complete to indicate their agreement.

**Project Description:**

This interview seeks to delve into the experiences of leaders within organizational settings. Its purpose is to gather essential insights to aid in developing items to measure leadership skills.

**Procedure and Risks:**

The researchers will interview through Microsoft Teams. At most, only two Research and Development Department interviewers will participate in the virtual meeting. The host interviewer will ask ten questions to tap into the participant’s experience as a company supervisor, manager, or executive. The researchers will ask the participants to answer as honestly as possible. The data collected from this interview will be a valuable source for developing an assessment tool for leadership skills.

Moreover, the researcher will request the participants' permission to record the virtual meeting. If consent is granted, the recording will facilitate precise transcription, a process expected to take approximately one month. Participants will be notified of the transcription's conclusion upon completion, and the recording will be promptly deleted. Alternatively, participants may opt out of recording the meeting, in which case the researcher will rely on notetaking. The interview is anticipated to last 45 minutes.

Participation in this interview is entirely voluntary. Participants can decline or withdraw from the interview at any time without consequence. If, at any point during the interview, a participant feels uneasy or experiences significant emotional discomfort, they have the right to withdraw without explaining. The researchers will respect this decision without question.

To withdraw from the interview before its commencement, participants may contact us via email at rnd@profilesasiapacific.com. Participants can inform the interviewer hosting the discussion during the interview if they wish to withdraw.

Rest assured, the researchers will uphold the anonymity of participants and ensure that the data collected will not compromise their privacy.

There are no known risks associated with participating in this interview.

**Benefits:**

The insights gained from this interview will be instrumental in developing an assessment tool tailored for supervisors and managers. This process ensures adherence to standard procedures for constructing situational judgment tests, guaranteeing accuracy in measuring leadership skills' difficulty levels. Following the publication of the assessment tool, the company retains ownership of the data. To safeguard participant anonymity, acknowledgments will refrain from including any demographic information.

**Confidentiality:**

The information collected during this phase will be handled with the utmost confidentiality. In cases where a participant's statements suggest an imminent threat to themselves or society, the researcher holds an ethical obligation to notify the relevant authorities. However, apart from such exceptional circumstances, the data obtained from the interview will be securely stored and accessible solely to the Research and Development Department.

**Confirmation:**

If the participant agrees to participate in this interview, please complete the consent form by ticking the box below. It should be noted that signing the consent form will signify the participant’s agreement to the terms discussed.

I consent to participate in this research project:

🞏 YES

🞏 NO
